# Inflamma-miRs in Aging and Breast Cancer: Are They Reliable Players?

**DOI:** 10.3389/fmed.2015.00085

**Published:** 2015-12-15

**Authors:** Cristina Sorina Cătană, George A. Calin, Ioana Berindan-Neagoe

**Affiliations:** ^1^Department of Biochemistry, Iuliu Hatieganu University of Medicine and Pharmacy, Cluj Napoca, Romania; ^2^Department of Experimental Therapeutics, MD Anderson Cancer Center, University of Texas, Houston, TX, USA; ^3^Non-Coding RNA Center, MD Anderson Cancer Center, University of Texas, Houston, TX, USA; ^4^Research Center for Functional Genomics, Biomedicine and Translational Medicine, Institute of Doctoral Studies, Iuliu Hatieganu University of Medicine and Pharmacy, Cluj-Napoca, Romania; ^5^Department of Experimental Pathology, Ion Chiricuta Institute of Oncology, Cluj Napoca, Romania

**Keywords:** inflammaging, inflamma-miRs, microRNAs, aging, breast cancer

## Abstract

Human aging is characterized by chronic low-grade inflammation known as “inflammaging.” Persistent low-level inflammation also plays a key role in all stages of breast cancer since “inflammaging” is the potential link between cancer and aging through NF-kB pathways highly influenced by specific miRs. Micro-RNAs (miRNAs) are small non-coding RNAs that negatively regulate gene expression at a posttranscriptional level. *Inflamma-miRs* have been implicated in the regulation of immune and inflammatory responses. Their abnormal expression contributes to the chronic pro-inflammatory status documented in normal aging and major age-related diseases (ARDs), inflammaging being a significant mortality risk factor in both cases. Nevertheless, the correct diagnosis of inflammaging is difficult to make and its hidden contribution to negative health outcomes remains unknown. This methodological work flow was aimed at defining crucial unanswered questions about inflammaging that can be used to clarify aging-related miRNAs in serum and cell lines as well as their targets, thus confirming their role in aging and breast cancer tumorigenesis. Moreover, we aim to highlight the links between the pro-inflammatory mechanism underlying the cancer and aging processes and the precise function of certain miRNAs in cellular senescence (CS). In addition, miRNAs and cancer genes represent the basis for new therapeutic findings indicating that both cancer and ARDs genes are possible candidates involved in CS and vice versa. Our goal is to obtain a focused review that could facilitate future approaches in the investigation of the mechanisms by which miRNAs control the aging process by acting as efficient ARDs inflammatory biomarkers. An understanding of the sources and modulation of inflamma-miRs along with the identification of their specific target genes could enhance their therapeutic potential.

## The Interconnected Nature of the Human Aging and the Role miRNAs in this Process

Aging is an inherently multifactorial process that is manifested within an organism at the genetic, molecular, cellular, organ, and system levels. Gene expression changes in aging at both protein and mRNA levels. An omnipresent feature of the aging process and most all age-related diseases (ARDs) is persistent inflammation. Research has recently shown the potential of Micro-RNAs (miRNAs) to modulate the development period, thus playing a major role in lifespan and the aging process. The spectrum of miRNAs is highly specific for different pathologies contributing to distinct patterns of gene expression (1–3).

The fact that a single miRNA has multiple targets is crucial for understanding the role of miRNAs in normal and pathological processes. The target mRNAs of a given miRNA could be predicted using homologies between the miRNA “seed” region and the complementary site on the target mRNA 3′-untranslated region (UTR) (1, 2). Furthermore, miRNAs are critical regulators of cellular processes such as cell division, differentiation, apoptosis, and senescence. The discovery of extracellular miRNAs enabled such small non-coding molecules to be used for monitoring both cancer and the biomarkers of aging. However, patients need additional investigations. In breast cancer, miRNAs have the potential to facilitate diagnosis and prognosis, predict the response to therapy, and act as therapeutic targets for miRNA-based replacement treatment (4–6). Age is known to be a major risk factor for several pathological conditions, including cancer, cardiovascular diseases, diabetes, and neurodegeneration (7). Breast cancer is the most frequent malignancy and the second cause of carcinoma-related death in women (5, 7).

Recent research demonstrated that miRNAs function in a multiple-to-multiple relationship with their specific target genes. Therefore, a certain miRNA can modulate expression of up to thousand mRNAs, and a specific mRNA can be coordinated by multiple miRNAs, suggesting that the interference of miRNAs in aging, inflammation, and cancer processes is complicated (1). Since miRNAs and their target mRNAs take effect cooperatively, it will be needed of a miRNAs network-based systems biology approach. Recently constructed, the human protein interaction network that consists of approximately 10,000 proteins has over 200,000 documented specific interactions (3). Moreover, the network of the aging process was proved to be a very efficient tool for integrating multiple strong links between the Human protein–protein interaction (PPI) network, the Human longevity network (HLN), and ARD transcriptional regulatory network nodes (proteins) (8).

microRNAs directly and indirectly connect senescence and tumorigenesis through HLN (1) (Table 1). Furthermore, from yeast to mouse, the pattern of evolutionary conservation of CS genes is almost identical to that of cancer-associated genes, this similarity being the result of the coevolution between these two processes. In addition, cellular senescence (CS) could be considered the tumor-suppresor mechanism of a molecular program that inexorably arrests cells at risk for malignant transformation. However, recent research reveals that senescent cells can also have detrimental effects on the tissue microenvironment. The most convincing of these effects is the acquisition of a senescence-associated secretory phenotype (SASP) that transforms senescent fibroblasts into pro-inflammatory cells able to promote tumor growth. CS genes, LAGs (longevity-associated) and cancer genes could be linked through miRNAs. Forty CS genes were validated as targets of 39 miRNAs. From these miRNAs many of them (including miR-21, miR-17, miR-29b) have targets involved in cancer, associated with other ARDs and longevity as well (1, 7, 9–11).

**Table 1:** **Common CS cancer-associated microRNAs and their target proteins found in HLN**.

miRNA	HLN targets	Reference
Let-7^b^	APP, NRAS, e-MYC^a^	(12–14)
miR-15^b^	Bcl-2, CCNE1	(15, 16)
miR-19a^b^	IMPDH, NPEPL1	(17)
miR-21^b^	Bcl-2, PDCD4, TPM1, TIMP3	(18–22)
miR-24^b^	Bim, Bcl-2	(23, 24)
miR-124	SphK1	(25)
miR-126^b^	SDF-1α	(26)
miR-145	CDH2, Oct4, MUC1	(27, 28)
miR-146a/b^b^	UHRF1	(29)
miR-155^b^	STAT3, SOCS1	(30, 31)
miR-214^b^	PTEN^a^	(32)
miR-221^b^	Slug (SNAI2)	(33)
miR-290^b^	Arid4b	(34)
miR-373	TXNIP, TRPS1, RABEP1, GRHL2, HIP1	(35)

*^a^Proteins with altered levels in age-related diseases*.

*^b^Breast cancer*.

*HLN, human longevity network; Bcl-2, B cell lymphoma2 (antiapoptotic protein); IMPDH1, inosine-5′-monophosphate dehydrogenase 1; NPEPL1, aminopeptidase like-1; PDCD4, programed cell death 4 (neoplastic transformation inhibitor); PTEN, phosphatase and tensin homolog; TPM1, tropomyosin 1 (alpha); TIMP3, TIMP metallopeptidase inhibitor 3; CCNE1, cyclin-E1; Bim, BH3-only domain-containing protein Bim, which positively regulates apoptosis; SphK1, sphingosine kinase 1 is an important enzyme encoded during neoplastic transformation; SDF-1α, stromal cell-derived factor 1-alpha; CDH2, N-cadherin; Oct4, OCT4 transcription factor (TF), MUC1, mucin1; UHRF1, ubiquitin-like, containing PHD and RING finger domains 1; STAT3, signal transducer and activator of transcription 3; SOCS1, suppressor of cytokine signaling 1; Slug, transcription factor; TXNIP, thioredoxin-interacting protein*.

**Figure 1:**
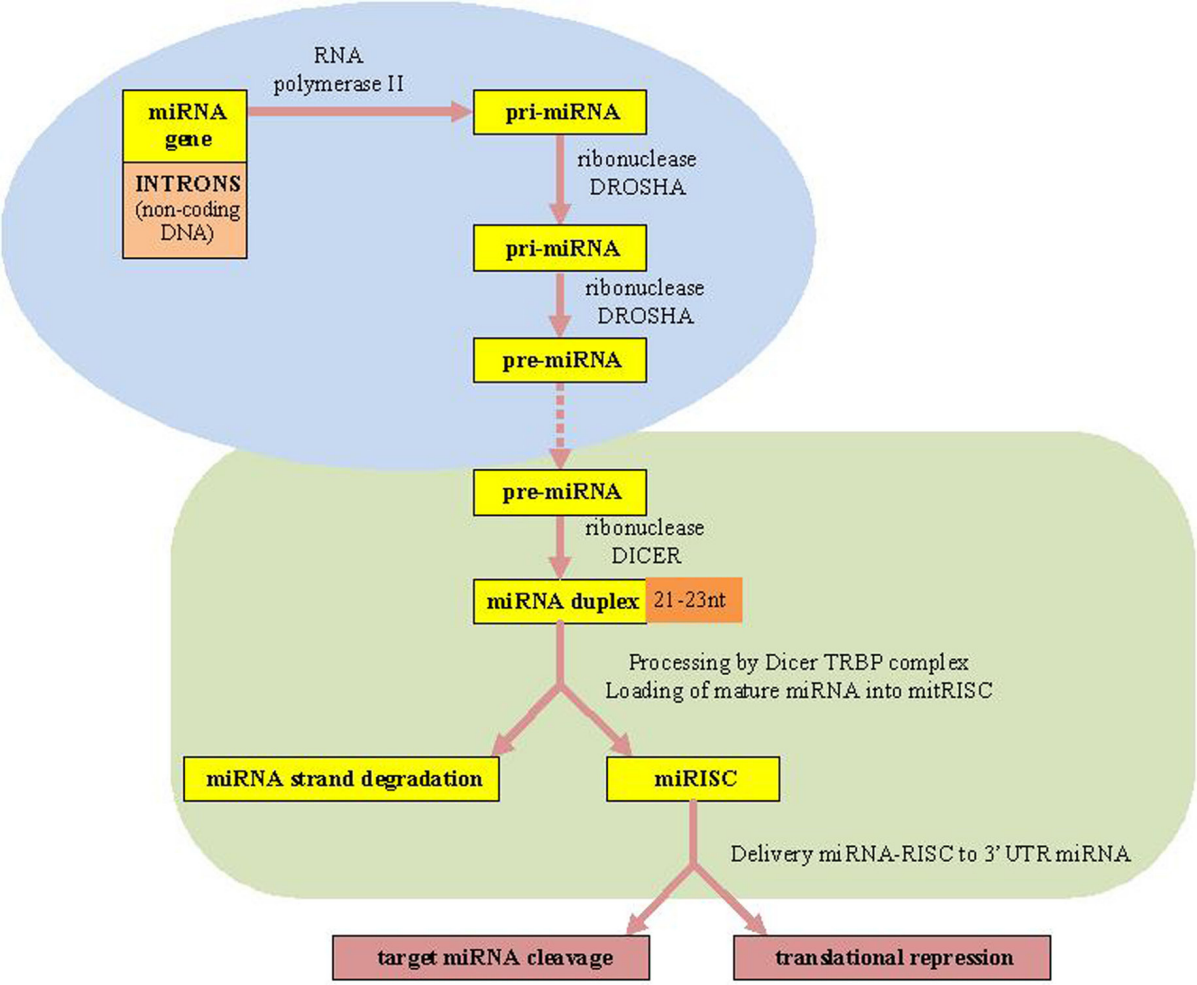
**microRNAs biogenesis**. miRNAs, microRNAs; RISC, RNA-induced silencing complex; TRBP, transactivating response (TAR) RNA-binding protein as a protein partner of human Dicer.

The canonical miRNAs biogenesis pathway starts in the nucleus (7). MiRNAs are firstly transcribed by RNA polymerase II (RNA Pol II) as an approximately 70-nucleotide (nt) long stem-loop primary structure named primary-miRNA transcripts, pri-miRNAs (long miRNA precursors), which are processed by DROSHA RNase III enzyme into a precursor to generate premiRNAs structure (10). Finally, the two strands of the duplex are separated from each other by the Dicer–TRBP complex. Next, the RNA-induced silencing complex (RISC), which also consists of the Argonaute protein and the target mRNA, is complementary bound by specific miRNAs. Consequently, the target mRNAs translation is repressed resulting in translational silencing (10, 11, 36) [Biogenesis of miRNAs is presented in Figure 1 adapted from Ref. (7, 10, 11)].

## microRNAs in Aging and Breast Cancer

microRNAs are unique due to their small size, which is approximately 22-nt-long. miRNAs control gene activity by interacting with the RNA that has been copied from DNA. There was no agreement on whether miRNAs were the “key” players in aging or not until 2008 (37). First, researchers were particularly interested in the inflammatory response and found an important role for a few miRNAs. They also showed that these small non-coding RNAs modulate the immune cell development as well as inflammation. In that research work, genetically engineered mice, blood stem cells, and *in vitro* techniques were used. Work was extended to examine the importance of miRNAs in the aging process, using microarrays in young and old mice (38). Subsequent research also revealed for the first time a signature of genotype-by-age changes in the circulating levels of miRNAs in the long-lived Ames mice (39).

The first evidence for the involvement of endogenous miRNAs in the control of lifespan was reported in 2005 (40, 41). The overexpression of miRNAs lin-4 led to an extended lifespan in *Caenorhabditis elegans* whereas its loss of it had the opposite effect (42). We also wish to focus on the important role of miRNAs in the immune function. Immunosenescence is “the key” to human aging, all aging-associated diseases (cancer, Alzheimer’s disease, metabolic diseases, and atherosclerosis) being caused by the chronic inflammation coordinated by oxidative stress and manifested by the increase in the level of proinflammatory cytokines, IL (interleukin)-1, IL-6, IL-17 coded by genes activated by the kappa B transcription factor (TF), NF-kB (nuclear factor kappa B) (43).

**Figure 2:**
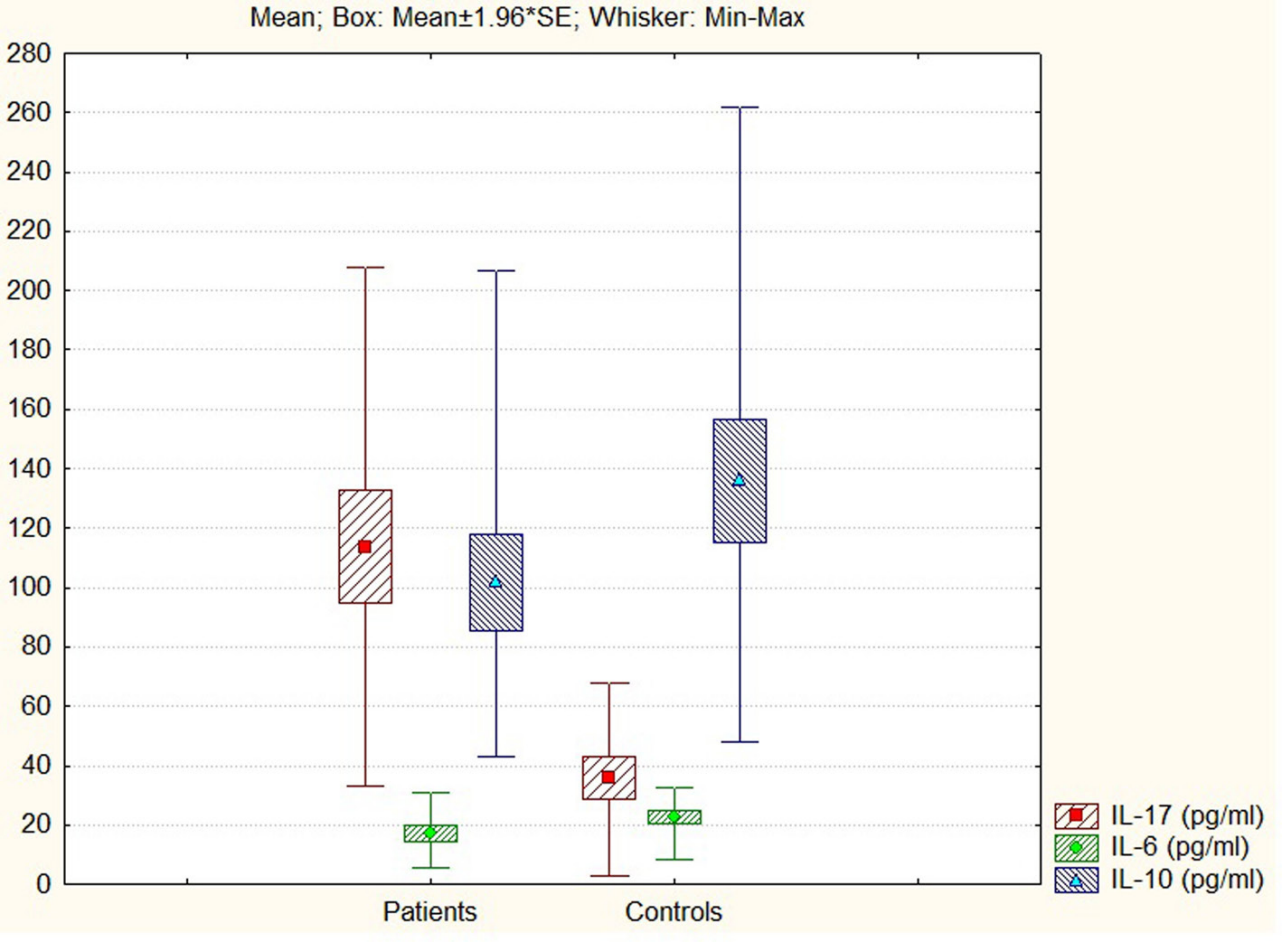
**IL-17, IL-6, and IL-10 levels in patients and controls**.

The comparative analysis between patients with ARDs (neurological or cardiovascular pathology) and controls revealed statistically significant differences in the anti (IL-10) and pro (IL-6, IL-17)-inflammatory investigated cytokines (44) (Figure 2).

Another reason to focus on miRNAs is that they are involved in chronic inflammation, a characteristic of aging and tumorigenesis. NF-κB is the master modulator of the pro-inflammatory status in these processes. A recent paper has shown that in mice, inhibiting one DNA-binding protein can revert aging skin to a pattern of gene activity characteristic to young skin. For example, inducible genetic blockade of NF-κB for 2 weeks in the epidermis of chronologically aged mice reverted the tissue characteristics and global gene expression programs to those of young mice. The chronic activation of this protein is indicative of an inflammatory condition, which strengthens the association between aging and inflammation and suggests that the examination of inflammatory miRNAs may be useful (45). Besides its major role in chronic inflammation, the downregulation of the NF-κB-signaling pathway could inhibit tumor cell growth in breast cancer (46).

The cellular participants involved in the pro-inflammatory status known as “inflammaging” cause senescence by inducing genotoxic stress and the SASP (1, 44, 47). These findings may be relevant for tumor growth, aging, and the senescence-activated inflammation responsible for the increased cancer incidence associated with aging. The inflammatory loop is fueled by SASP. Multiple doses of rapamycin, a promising therapeutic agent with both anti-tumor and immunosuppressive properties selectively splits the cascade of pro-inflammatory events associated with CS (48). Aging is plastic, as it can be shaped by adequate interventions such as specific miRNAs, which could be active components of SASP (49). This fact might contribute to the disease-free phenotype known as “Healthy Aging” (50, 51).

Researchers identified miRNAs that can be associated with cancer (oncomiRs), inflammation (inflamma-miRs), and aging/senescence (SA-miRs). Three of them, namely, miR-21, miR-126, and miR-146a and their target mRNAs belong to the NF-kB pathway, which is the master modulator of the pro-inflammatory status in ARDs (49, 52). MiR-126 and miR-126^⋆^, a miRNA pair derived from a single precursor, repress recruitment of mesenchymal stem cells and inflammatory monocytes to inhibit breast cancer metastasis, which demonstrates a correlation between miR-126/126^⋆^ downregulation and poor metastasis-free survival in breast cancer patients (26).

A senescent cell phenotype reduced the expression of proliferation-stimulating/apoptosis-suppressing miR-21, miR-214, and miR-92 and increased the expression of tumor suppressors and apoptotic markers; inflammation-repressing miR-126 was reduced, whereas inflammatory proteins had a higher level in senescent human aortic endothelial cells (53). For example, high levels of miR-21 occur in invasive breast tumors. There are two tumor suppressors among the miR-21 targets: tropomyosin I (TPMI) and phosphatase and tensin homolog (PTEN), plus the proteins involved in suppression, invasion, and metastasis: programed cell death 4 protein (PDCD4) and maspin (54–56).

In cancer cells, all these proteins are inhibited by high levels of miR-21, while inhibition of this miRNA has the opposite effect of decreasing tumor cell growth, migration, and invasion (57). It has recently been demonstrated that miR-146a plays an important role in the modulation of the innate immune response (58). Several studies have shown the relevance of the upregulation of NF-kB/miR-146a in breast cancer. In addition, by counteracting the pro-inflammatory effects of CS, miR-146 provides anti-inflammatory effects and general suppressive action (52).

## microRNAs in Breast Cancer: oncomiRs and Tumor Suppressor microRNAs

Normal cells must acquire several characteristics in order to become a tumor. Furthermore, cancerous cells thrive if they maintain a proliferative status, survive despite strict environmental conditions, induce local angiogenesis, invade other tissues, metastasize and avoid being recognized by the immune system (59). Non-coding small miRNAs play an important role in cancer (60–62). The association between miRNAs and breast cancer has been recognized since 2005, including multiple functions such as suppression of tumorigenesis, promotion or inhibition of metastasis, and sensitivity or resistance to chemotherapy. According to the role of miRNAs in cancer cell phenotypes, some are oncogenic (oncomiRs) and others are tumor-suppressive (TS-miRNAs), depending on the cellular context and on their specific targets in each cellular event (62, 63).

Underexpressed miRNAs in cancers, such as the let-7 family members (TS-miRs) function as tumor suppressor genes. They also induce p53 mediated apoptosis, inhibit the cell cycle and cause breast cancer resistance to tamoxifen. Moreover, experiments carried out between 2008 and 2013 suggest that let-7 based therapy in breast cancer could be available in the near future, especially for drug resistant and estrogen-positive metastatic variants (13). In addition, let-7 is a well-known enhancer of CS (14).

Conversely, overexpressed miRNAs in cancer such as miR-17-92 may function as oncogenes and promote cancer development by negatively regulating tumor suppressor genes that control apoptosis or cell differentiation. In breast cancer, excellent information coverage on certain miRNAs named oncomiRs was also provided (64, 65) [Table 2; (5, 7, 18–22, 66–71)].

**Table 2:** **Breast cancer oncomiRs and their target proteins**.

OncomiRs	Breast cancer-related proteins encoded by mRNAs that are oncomiRs targets	Reference
miR-21	Bcl-2, PDCD4, TPM1, TIMP3	(18–22)
miR-155	Caspase 3, SOCS1	(5, 66)
miR-27a	ZBTB10, FOXO1	(67, 68)
miR-96	FOXO1	(68)
miR-182	FOXO1	(68)
miR-128a	TGF-βR1	(69)
miR-10b	Tiam1, TWIST, HOXD10, E-cadherin	(5, 70)
miR-9	E-cadherin	(5, 71)
miR-373	CD44	(7)
miR-520c	CD44	(7)

*Bcl-2, B cell lymphoma2 (antiapoptotic protein); PDCD4, programed cell death 4 (neoplastic transformation inhibitor); TPM1, tropomyosin 1 (alpha); PTEN, phosphatase and tensin homolog; TIMP3, TIMP metallopeptidase inhibitor 3; SOCS1, tumor suppressor gene suppressor of cytokine signaling; ZBTB10, zinc finger and BTB domain containing 10; FOXO1, Forkhead box protein O1; TGF-*β*R1, transforming growth factor-*β* type 1 receptor; Tiam1, T lymphoma invasion and metastasis; TWIST, twist-related protein; HOXD10, homeobox D10*.

Tumor suppressor miRNAs are extremely effective in breast cancer progression because they suppress cell growth, proliferation, angiogenesis, invasion, and metastasis. They also enhance cell death and immune recognition, as well as cancer therapy. For most of the above processes, TS-miRNAs are downregulated in cancer tissues. Upon re-expression they suppress tumorigenesis, including proliferation, apoptosis, and migration (72) [Table 3; (5, 7, 73–98)].

**Table 3:** **TS-miRNAs involved in breast cancer**.

TS-miRNAs	Breast cancer-related proteins encoded by mRNAs that are TS-miRNAs targets	Reference
**Cell growth and proliferation**		
miR-34a	Bcl-2, SirT1	(73)
miR-17-5p	AIB1	(74)
miR-125b	Ets 1, Bcl-2	(75, 76)
miR-128	EGFR, PDGFRα	(77)
miR-125b	EPO, EPOR, ENPEP, CK2-α, CCNJ, MEGF9	(7)
**Cell survival**		
miR-34a	Bcl-2, SirT1, BIRC3, DcR3, c-Met, Notch-1, Notch-2, Cyclin D1, Cyclin E2, Cdk4, Cdk6, E2F	(73, 78, 79)
**Angiogenesis**		
miR-145	VEGF-A, N-Ras, p70s6K1	(80, 81)
miR-519c	HIF-1α, HuR	(82–87)
miR-340	c-Met	(88)
miR-126	IGFBP2, MERTK, PITPNC1	(7)
**Suppressors of immune recognition**		
miR-322	Galectin-3	(89)
miR-93	Genes of the TGF-β and/STAT3 pathway	(90)
**Invasion and metastasis, EMT**		
Let-7 families	H-ras, HMGA2, PAK1, DIAPH2, RDX, ITGB8	(5, 91)
miR-200 families	ZEB2	(5)
miR-205	ZEB1, ZEB2	(5, 92)
miR-335	SOX4	(5)
miR-340	c-Met	(88)
miR-34a	c-Met	(93)
miR-145	VEGF, N-Ras	(80)
miR-183	Villin 2 (Ezrin)	(94)
miR-19a	Fra-1	(5, 95)
miR-17-92	Mekk2	(7, 96, 97)
miR-206	Cyclin D2, Cx43	(7)
miR-146b	NFkB, STAT3	(7, 98)
miR-31	RhoA, WAVE3	(7)

*Bcl-2, B cell lymphoma2; SirT1, sirtuin 1; AIB1, amplified in breast cancer; Ets 1, protooncogene; EGFR, epidermal growth factor receptor; PDGFRα, platelet derived growth factor; BIRC3, baculoviral IAP repeat-containing 3; DcR3 (decoy receptor 3), c-Met, Notch-1 and Notch-2 are pro-cell-survival factors; Cyclin D1, Cyclin E2, Cdk4, Cdk6, E2F-cell cycle regulators; VEGF-A, vascular endothelial growth factor-A; N-Ras, tumor suppressors; p70s6K1, serine/threonine kinase; HIF-1α, hypoxia inducible factor-1 alpha; HuR, Hu-antigen R; c-Met, hepatocyte growth factor receptor; VEGF, vascular endothelial growth factor; H-ras, transforming protein p21; HMGA2, high-mobility group AT-hook 2; PAK1, serine/threonine-protein kinase 1; DIAPH2, protein diaphanous homolog 2; RDX, radixin; ITGB8, integrin *β*-8; ZEB1, zinc finger E-box-binding homeobox 1; ZEB2, zinc finger E-box-binding homeobox 2; EMT, epithelial to mesenchymal transition, Fra-1, Fos-related antigen 1; EPO, erytropoietin; EPOR, erytropoietin receptor; ENPEP, glutamylaminopeptidase or aminopeptidase A; CK2-α, casein kinase 2-alpha; CCNJ, cyclin J; MEGF9, multiple EGF-like domains 9; Mekk2, mitogen activated protein kinase kinase kinase 2; Cx43, connexin 43; STAT3, signal transducer and activator of transcription 3; NFkB, nuclear factor kappa B; RhoA, Ras homolog gene family; WAVE3, WAS protein family, member 3; IGFBP2, insulin-like growth factor-binding protein 2; MERTK, c-Mer tyrosine kinase; PITPNC1, phosphatidylinositol transfer protein, cytoplasmic 1*.

microRNAs such as TS-miRNAs (let-7, miR-15a/16, and miR 143/145) act as tumor suppressors while miR-21, miR-17-92, and miR-155 act as oncogenes by suppressing and promoting tumorigenesis. Highly specific miRNAs also control the development of the metastatic phenotype and are present in different types of cancer (72).

In a certain stage of the same cancer, they are used as a marker for diagnosis, prognosis, and therapy response. In invasive breast tumor, two miR-21 targets mentioned before (TPMI, PTEN) are inhibited by high levels of miR-21, while inhibition of this miRNA has the opposite effect of decreasing tumor cell growth, migration and invasion (19, 20, 55, 57).

## Inflamma-miRNAs Modulation in Cancer and Aging

Interestingly, all ARDs, including cancer share inflammation as a common denominator, CS being at the basis of the chronic inflammatory state in aging (99). Many individual miRNAs identified in humans target “key” proteins involved in aging and cancer, thus demonstrating their crucial role in aging and breast cancer tumorigenesis. For example, miR-146b increase with age and repress the senescence-associated proinflammatory cytokines IL-6 and IL-8 Activator of transcription 3 (STAT3) and interleukin-6 (IL-6)-mediated signal transducer could be mechanisms by which chronic inflammation contributes to breast cancer such as a common oncogenic event. The gene encoding the TS miRNA, miR-146b is a direct STAT3 target gene, its expression being decreased in tumor cells but increased in normal breast epithelial cells. Moreover, miR-146b inhibits NF-κB-dependent production of IL-6, subsequent STAT3 activation, and IL-6/STAT3-driven invasion and migration in breast cancer cells. Therefore, higher expression of miR-146b was positively correlated with patient survival in breast cancer subtypes with increased IL6 expression and STAT3 phosphorylation. The use of antagomirs directed against breast tumor cells represents a new and highly important therapeutic method (98, 100).

MiR-21 promoter contains binding sites for the TF STAT-3 activated by IL-6 signaling pathway (37). In the elderly, an increased production of proinflammatory cytokines such as IL-6 was observed, which proves the link between tumorigenesis and the aging process; miR-21 was also found elevated in other ARDs such as hypertrophic heart, neointimal formation and Alzheimer’s disease. High levels of pro-inflammatory molecules found in centenarians were offset by large amounts of anti-inflammatory molecules such as TGF-β and IL-10. Besides CDK (cyclin-dependent kinase) regulators driving cell cycle progression in all eukaryotes, new strategies for controlling the NF-kB related inflammation pathway in pathological aging should be used (101, 102).

MiR-519 represses the production of HuR, an RNA-binding protein very abundantly found in tumor cells and less expressed in untransformed cells, while the overexpression of HuR delays the senescent phenotype (83).

MiR-155, which is only one example, out of the many miRNAs could be considered a bridge between inflammation and breast cancer. The overexpression of miR-155 in breast cancer cells leads to constitutive activation of signal transducer and activator of transcription 3 (STAT3) through the Janus-activated kinase (JAK) pathway, and stimulation of breast cancer cells by the inflammatory cytokines IFN-γ and interleukin-6 (IL-6), lipopolysaccharide (LPS), and polyriboinosinic: polyribocytidylic acid [poly(I:C)] significantly upregulates *mir-155* expression. In addition, the suppressor of cytokine signaling 1 (socs1) is a new target of miR-155 in breast cancer cells, thus contributing as a consequence to the constitutive STAT3 activation. The cross talk between miR-155, SOCS1, and STAT3 signaling may provide a new mechanism for inflammation-associated tumorigenesis, which suggests that miR-155 and SOCS1 could potentially be used in cancer therapy (30, 31).

Furthermore, serum miR-155 could be a potential biomarker for differentiating between cancer patients and healthy subjects, as well as an indicator of treatment response. Low levels of miR-155 were also observed after surgery and chemotherapy (103, 104). In addition, serum miR-155 is particularly upregulated in more advanced cancer stages than in low-grade breast tumor (105).

The overexpression of miR-34a, a TS-miRNA that represses the production of Bcl-2 and SirT1 (two proteins displaying high levels of expression in breast tumors, which are involved in cell growth and proliferation) induces the senescence (the phenomenon by which normal diploid cells cease to divide) of cancer cells. This miR impairs angiogenesis by induction of senescence via SirT. Higher miR-34c was observed in senescent cells and also in the blood of breast cancer women, the reduced expression of miR-34c being particularly important for progression to the most advanced stages. It also controls the NF *NF*-*kB* and could be considered an inflamma-miR (105). Let-7 miRNAs are also upregulated in senescent cells, thus suppressing tumor growth and contributing to the aging process (52).

MiR-195, which is involved in the activation of *NF*-*kB*, is highly increased in the blood of breast cancer patients and in CS. The abrogation of the miR-195 expression is a promising therapy in elderly patients. Therefore, miR-195 could serve as an inflammatory biomarker in ARDs (105, 106). Based on the finding that certain miRNAs decreased tumorigenesis, and it was proposed that the coordinated action of upregulated-senescence inflamma-miRs could block cancerous cell growth by reducing oncogenes and tumor promoters’ levels. Their regulation chelp treat ARDs (83, 107).

**Figure 3:**
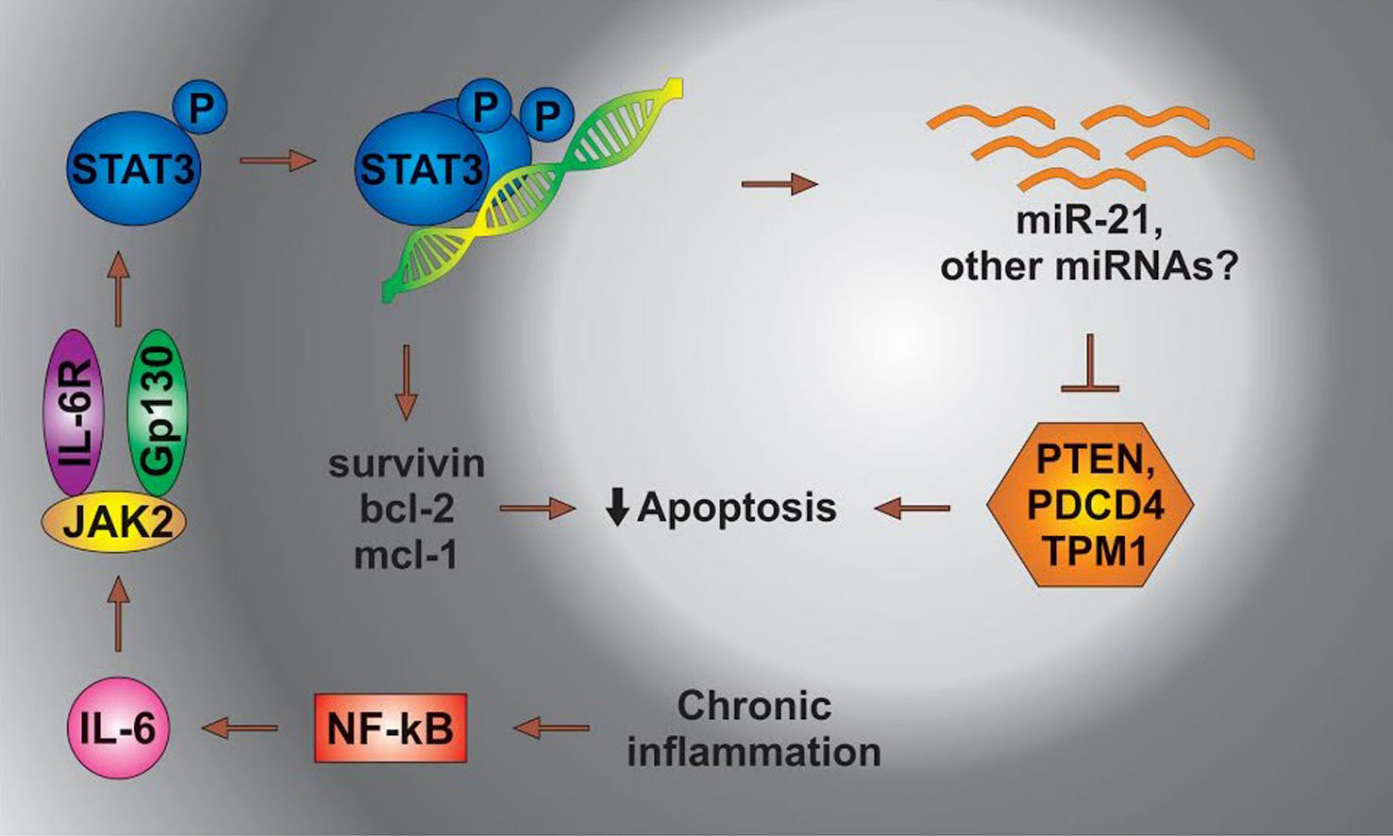
**The status of miRs in chronic inflammation**. STAT3, signal transducer and activator of transcription 3; NFkB, nuclear factor kappa; PDCD4, programed cell death 4 (neoplastic transformation inhibitor); TPM1, tropomyosin 1 (alpha); PTEN, phosphatase and tensin homolog B; Bcl-2, B cell lymphoma 2; mcl-1, myeloid cell leukemia-1; JAK 2, Janus kinase-2 (the Janus family of tyrosine kinase plays an essential role in coupling cytokine receptors to downstream intracellular signaling pathways).

IL-6 leads to the activation of Stat3, which then dimerizes and binds to its cognate sites located in the regulatory regions of genes. The Stat3-mediated transcription of several antiapoptotic genes contributes to the survival of cancer cells. In a separate mechanism, Stat3 directs the expression of miR-21, resulting in the suppression of apoptosis possibly through the inhibition of TPM1 and/or other proteins [Figure 3 adapted from Ref. (108)].

The *inflammaging* phenotype results from age-related cell and tissue adaptation/remodeling interacting with the genetic/epigenetic background. This is a complex phenotype involving not only innate but also adaptive immunity and affecting a range of tissues and organs such as the gut, liver, muscle, and brain. Importantly, *inflammaging* appears to be accelerated in a variety of age-associated diseases. Tissue and circulating *inflamma-miRs* could restrain the activity of the senescent cell secretome and check the destruction induced by the activation of the inflammatory response (109).

*Inflamma-miRs* have been involved in the regulation of the immune and inflammatory response, and their abnormal expression may contribute to the low-level chronic inflammation that has been documented both in normal aging and in the major ARDs (109).

Circulating *inflamma-miRs* could thus have diagnostic/prognostic relevance in human diseases with a common inflammatory background, such as cancer. Recent studies have shown upregulation of *inflamma-miRs* in the circulation of healthy elderly and chronically ill old individuals: the increase is less pronounced in centenarians and greater in patients with ARDs. It is generally accepted that the main sources of circulating *inflamma-miRs* in aging and ARDs are immunity circulating/tissue cells and endothelial circulating/resident cells. Inflammatory stimulation and cell senescence can induce and perpetuate systemic inflammation over time, by inducing the upregulation of *inflamma-miRs* through the excessive activation of inflammatory pathways (109, 112) [Table 4; (105, 106, 109–112)].

**Table 4:** **Circulating inflamma-miRs in breast cancer and cellular senescence**.

Circulating inflamma-miRs	Samples	Reference
miR-21	Serum	(102, 110, 111)
miR-126	Plasma/serum	(109)
miR-146a	Plasma	(109)
miR-155	Serum	(110)
Let-7a	Plasma/serum	(97, 105)
miR-34a	Plasma/serum	(105)
miR-195	Plasma/serum	(105, 106)

## Concluding Remarks

Circulating miR-21, miR-34, miR-126, miR-195, let-7a, miR-146a, and miR-155 could be considered as inflamma-miRs that modulate the NF-kB signaling pathway in aging, inflammation, and breast cancer. The similar features of miRNAs in cancer and aging elucidate the functions and therapeutic implications of these common small RNAs. Interestingly, a number of such circulating miRNAs seem to be promising biomarkers for major ARDs that share a common chronic, low-level pro-inflammatory status, such as breast cancer and other ARDs.

A better understanding of the sources and modulation of inflamma-miRs, along with the identification of their specific target genes may enhance their therapeutic potential. New drug development and biological understanding of the inflammatory processes need to be improved. The seven miRs are promising targets as they can be an extremely selective and effective therapeutic strategy against aging and breast cancer.

## Author Contributions

IB-N and CC initiated this review, searched the literature, and contributed largely to the writing of the manuscript. GC contributed with his own opinions to this review, surveyed, and advised the literature search. IB-N revised all the versions of the manuscript and gave the final approval of the version to be published. IB-N, GC, and CC drafted the article and revised it critically for important intellectual content.

## Conflict of Interest Statement

The authors declare that the research was conducted in the absence of any commercial or financial relationships that could be construed as a potential conflict of interest.
